# Laboratory Tests of Metrological Characteristics of a Non-Repetitive Low-Cost Mobile Handheld Laser Scanner

**DOI:** 10.3390/s24186010

**Published:** 2024-09-17

**Authors:** Bartosz Mitka, Przemysław Klapa, Pelagia Gawronek

**Affiliations:** 1 Department of Agricultural Land Surveying, Cadastre and Photogrammetry, University of Agriculture in Krakow, 21 Mickiewicza Ave., 31-120 Krakow, Poland; bartosz.mitka@urk.edu.pl; 2 Department of Land Surveying, University of Agriculture in Krakow, 21 Mickiewicza Ave., 31-120 Krakow, Poland; pelagia.gawronek@urk.edu.pl

**Keywords:** MLS, LiDAR, non-repetitive, low-cost scanning, scanning accuracy and precision, measuring instrument

## Abstract

The popularity of mobile laser scanning systems as a surveying tool is growing among construction contractors, architects, land surveyors, and urban planners. The user-friendliness and rapid capture of precise and complete data on places and objects make them serious competitors for traditional surveying approaches. Considering the low cost and constantly improving availability of Mobile Laser Scanning (MLS), mainly handheld surveying tools, the measurement possibilities seem unlimited. We conducted a comprehensive investigation into the quality and accuracy of a point cloud generated by a recently marketed low-cost mobile surveying system, the MandEye MLS. The purpose of the study is to conduct exhaustive laboratory tests to determine the actual metrological characteristics of the device. The test facility was the surveying laboratory of the University of Agriculture in Kraków. The results of the MLS measurements (dynamic and static) were juxtaposed with a reference base, a geometric system of reference points in the laboratory, and in relation to a reference point cloud from a higher-class laser scanner: Leica ScanStation P40 TLS. The Authors verified the geometry of the point cloud, technical parameters, and data structure, as well as whether it can be used for surveying and mapping objects by assessing the point cloud density, noise and measurement errors, and detectability of objects in the cloud.

## 1. Introduction: Research on Low-Cost Mobile Scanners

Recent years saw the commercial emergence of smaller and inexpensive solutions primarily for autonomous vehicles and robotics as an offshoot of precise, high-resolution scanning systems dedicated to spatial surveys. Their growth is driven by the increasing demand for low-cost systems that can provide volumetric spatial information in real time. These devices are increasingly common in terrestrial static and dynamic mapping systems as primary sensors or on UAVs. More and more authors publish the results of their tests of such instruments regarding their measurement characteristics, such as accuracy, data density, noise, or artifacts. For example, Ortiz Arteaga and colleagues [1] presented results on the performance of a low-cost automotive LiDAR, Livox Mid-40 (Livox, Livox Technology Company Limited Co., Hong Kong https://www.livoxtech.com). Their objective was to investigate the quality of the sensor for ranging, repeatability, and accuracy. Their results indicated that the device could be used for reality capture purposes, such as to generate rough as-built plans or volume calculations. The reference values for accuracy tests came from Leica RTC 360 (Leica Geosystems, Hexagon, Germany). Other sources provide insight into the calibration and stability of such scanners. For example, Glennie et al. [2] contributed such data for Velodyne VLP-16. A work by Glennie and Hartzell [3], ‘Accuracy assessment and calibration of low-cost autonomous lidar sensors’, provides a detailed methodology for the metrological evaluation of low-cost mobile laser scanners, with Livox Mid-40 and Ouster OS1-64 as examples. These authors also employed reference data from a terrestrial laser scanner (Riegl VZ-2000; RIEGL Laser Measurement Systems GmbH, Austria). Many other publications tackle the issues of the calibration and integration of low-cost laser scanners with inertial modules and other sensors concerning their accuracy and adjustment of trajectory [4,5] or the accuracy of volumetric data [6,7]. Other authors discussed in depth the combination of Simultaneous Localization and Mapping (SLAM) algorithms and low-cost scanners with a small Field of View (FOV) [8,9].

An assessment of the accuracy and precision of laser scanners (regardless of the mode) has to consider the numerous sources of errors that affect the outcomes [10]. The first group of accuracy factors is the problem of ranging with laser rangefinders. The ranging system in laser scanners determines the critical parameters of these devices, namely, the range, accuracy, scanning time, and noise. Scanning systems employ three categories of ranging methods: distance measurement through triangulation, methods based on time of flight, and interferometric methods [11,12,13]. Laser scanners’ time-of-flight-ranging systems draw on solutions found in electro-optical rangefinders and total stations. The methods used in these devices take advantage of the property of light waves travelling through the air at a known speed. The distance is calculated by measuring the time it takes an emitted signal to make the round trip to the measured object and back [14]. This time can be measured with two methods that use two forms of the signal: direct (pulse) and indirect (phase). The direct method involves a direct measurement of the travel time of a discrete pulse [15], while the indirect method measures the time of a continuous sinusoidal light wave. The precision of the direct method is affected by the accuracy of the time measurement, which needs to be high considering the speed of light. The accuracy can be improved by employing a series of pulses emitted at a constant frequency [15,16,17]. Apart from the time measurement accuracy, the reliability of the direct ranging method is determined by how the return signal is detected [18,19]. The indirect method involves the emission and reception of a continuous sinusoidal wave (CW) signal. A comparison of the received, reflected signal with the emitted reference wave can be used to measure the time indirectly. The signal can be modulated in several ways: through amplitude modulation (AM), frequency modulation (FM), polarization modulation, or pseudo-noise modulation [12,18,20]. Phase-based laser scanners modulate the light-wave beam by changing its phase and measure the difference between the outgoing and incoming signal phases or the phase shift, using phase modulation of the laser light to determine distance [16,17]. The direct method facilitates a longer range at the cost of limited accuracy. Despite precise time-measuring devices, the accuracy of pulse laser scanners is much lower than that of phase-based scanners. They are also slower. The wavelength seriously limits the phase ranging method, which determines the maximum scanning range and is prone to interference: points outside the maximum range are assigned a wrong distance.

Another accuracy factor is the scanner design errors that violate primary geometrical requirements for measuring instruments. Considering that most laser scanners have rotating mirrors for beam deflection, a slight angular difference in the mirror system can result in a significant error in point cloud coordinates, which grows as a function of distance to the scanner. The angular accuracy depends on the mirror positioning error and the angular accuracy of the measuring device. The problem of the angular accuracy of laser scanners is discussed as a specific case of laser scanner positioning [20]. The next type of error of the instrument is connected with the beam spot size, which directly influences the severity of the edge effect [21]. The laser beam is highly coherent, monochromatic, and directional. Additionally, its divergence angle does not exceed several milliradians. Due to monochromaticity, the laser emits a very narrow wavelength, yielding high accuracy, particularly for phase-based measurements. Thanks to high directionality, the laser beam diameter increases only slightly with distance [22]. The beam spot diameter is a critical parameter of laser scanning. It grows linearly with the distance. Spot size change is connected with how a Gaussian beam is propagated because the laser beam is considered Gaussian [23]. At the same time, as the incidence angle of the laser beam (from normal to the surface) increases, the spot diameter grows, and the spot becomes oval and elongated. These are the direct causes of the edge effect. The edge effect was discussed by Boehler et al. [24], Cosarca et al. [10], and Klapa and Mitka [21], who demonstrated that its impact on point positioning accuracy cannot be overstated.

Another source of errors dragging point positioning accuracy down is factors inherent to the measured object, particularly its reflective properties, beam dispersion, and incidence angle [10]. The intensity of the reflected pulse depends on the reflective properties of the object’s material, affected by color, coarseness, temperature, and moisture content [25]. Boehler et al. [24] proposed an empirical method for determining the reflective properties. The problem was also investigated by Kersten et al. [26], Pesci et al. [25], and Cosarca et al. [10]. Intensity also depends on the scanned material’s humidity because the water content can affect wave reflection [10].

The incidence angle is also a factor, depending on the scanner’s position relative to the object and the object’s geometry. As the incidence angle increases, the spot size grows, and the positioning accuracy deteriorates, as does the intensity of the return signal. Clark and Robson [27] concluded that the optimal scanner–object arrangement is when the laser beam is perpendicular to the scanned surface. Properly planning the number and density of stations can help eliminate incidence-related errors. At the same time, the larger the incidence angle, the lower the point cloud density. Clark et al. [27] noted an increase in noise for angles over 75 degrees. Beam scattering is yet another determinant of data quality. According to the law of reflection, the incidence angle is equal to the reflection angle, and the incidence angle, reflection angle, and the normal to the surface lie in the same plane. This is true only for perfectly smooth surfaces; otherwise, the laser beam is scattered [27].

The third group of measurement accuracy factors are environmental circumstances. These include temperature, humidity, ambient lighting, and dust [10,27]. Changes in these parameters affect the refractive index. The engineer should always observe the manufacturer’s working temperature recommendations during scanning. The last group is procedural errors due to poor scan planning. These include selecting an inappropriate measurement method and scanner or poor station position planning [28]. Before every scan, the engineer needs to analyze the object’s geometry, color, and texture because these parameters directly affect the positioning accuracy [29].

Despite its tremendous potential, MLS still requires human intervention and post-processing to yield the desired results. Nevertheless, mobile mapping systems can address some problems of traditional static methods, particularly concerning time efficiency, which refers to the significantly reduced time required for data collection, scanning, and post-processing, as confirmed in tests where the two were juxtaposed [30]. Although data inpainting is necessary in most cases to obtain a reliable result, which demonstrates the technical and methodological limitations of laser scanners [31,32], manual mobile scanners are gaining popularity thanks to their speed and accuracy. Apart from construction, mobile laser scanning is widely used in various domains, such as cultural heritage and archeology, environmental monitoring, forestry, agriculture, and many more [31].

This article aims to determine the metrological characteristics of the low-cost mobile laser scanner MandEye (MANDALA ROBOTICS—Janusz Bedkowski, Poland, https://mandalarobotics.com). The mobile handheld laser scanner MandEye is a relatively new solution marketed first in 2023. The hardware and software concept was presented by the creator, J. Będkowski, in ‘Open source, open hardware hand-held mobile mapping system for large scale surveys’ [33]. The open-hardware project (https://github.com/JanuszBedkowski/mandeye_controller (accessed on 15 May 2024) [34] provides all the necessary information on how to construct a handheld mobile mapping system that records 3D and IMU (Inertial Measurement Unit) data. The open-source project (https://github.com/MapsHD/HDMapping (accessed on 29 May 2024)) [35] consists of three components:

- LiDAR odometry (step 1)
- multi-view TLS (Terrestrial Laser Scanner) registration (step 2)
- multi-session registration (step 3).

The metrological characteristics tests involved measurements under laboratory conditions using reference objects and a reference base from data generated by a higher-accuracy laser scanner, Leica ScanStation P40 TLS (Leica Geosystems, Hexagon, Germany). The tests focused on the metrological characteristics and measurement properties of a point cloud yielded by MandEye in the static and dynamic modes. In addition to the measurement, data processing, and analyses for determining the device’s measurement parameters, we also set out to verify whether using it in land surveying is possible. This was accomplished by assessing the correctness, accuracy, and stability of object detectability in a MandEye MLS point cloud against the reference base.

The article is structured into four primary parts. The introduction, along with a review of past tests of handheld MLS accuracy and characteristics of laser scanner measurement errors, is followed by the second part, which presents research methods and measuring tools used in the study, together with the research diagram. The third part presents and discusses the results in the context of outcomes reported in the literature. The last part is a summary with conclusions from the tests and accuracy analyses. It provides insights into the potential practical use of the handheld low-cost mobile laser scanner MandEye.

## 2. Materials and Methods

### 2.1. Laboratory Tests—Test Field

**Figure 1:**
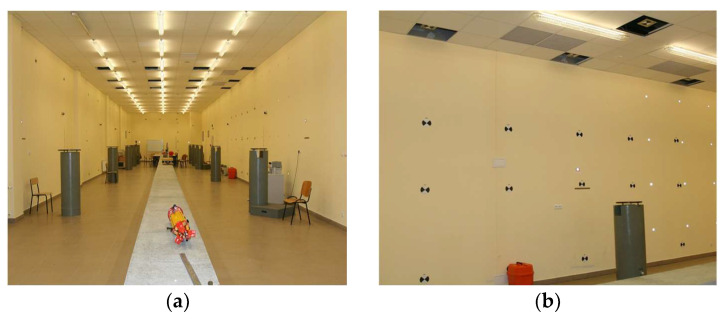
The laboratory: (**a**) general view, (**b**) survey points.

The surveying laboratory of the Faculty of Environmental Engineering and Land Surveying, University of Agriculture in Kraków, is located in the basement of the faculty building at 253c Balicka Street in Kraków, Poland. The laboratory is 37.40 m long, 6.78 m wide, and 4.22 m high. The temperature, atmospheric pressure, and air humidity in the room are monitored by a digital weather station. There are 14 concrete pedestals in the room in two rows of seven. There are steel leveled heads on their tops for the forced centering of instruments and targets (including high-precision spheres for laser scanners). The pedestals are evenly distributed along the length of the room. Each head has a central point and three or seven eccentric seats for measuring instruments (Figure 1a). All the heads have been leveled, and their heights have been determined by adjusted precision leveling. Moreover, all the pedestals have height marks to monitor the height frame of the entire room. The XY coordinates of the central and eccentric points of the heads have been precisely determined (multiple adjusted surveys with a precise total station) for the local frame and provide the reference frame for laboratory research.

In addition, there are survey points on the laboratory walls and ceiling (Figure 1b), namely:

- black and white targets for laser scanners;
- Leica GMP104 prisms;
- pieces of reflective film.

The local laboratory XYZ coordinates of all the points have been determined with the survey base. The points on the heads, walls, and ceiling, whose XYZ coordinates have been precisely determined, constitute the volumetric calibration field for research on the following:

- the measurement accuracy of laser scanners and surveying instruments;
- the relative orientation of devices in complex measurement systems;
- the absolute orientation of mobile measuring systems.

Researchers can obtain reliable results for various measuring devices by selecting the right tie-up and control points, applying dedicated surveying and post-processing methods for each tested device, and using auxiliary measured objects and control devices in the laboratory.

**Figure 2:**
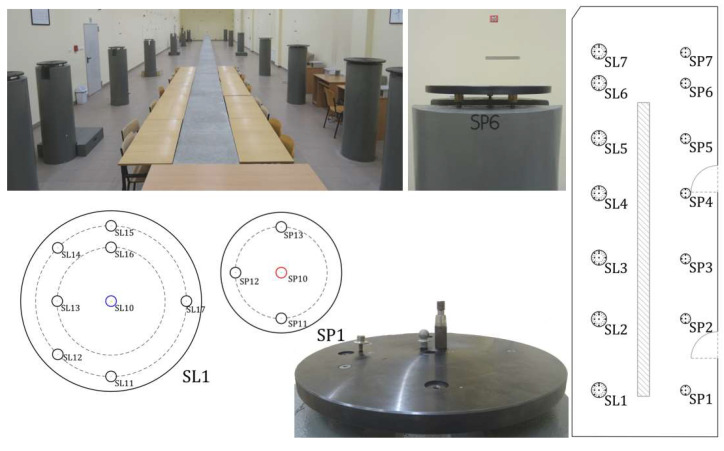
Test laboratory.

The laboratory tests of the low-cost manual laser scanner consisted of several sequences, including static and dynamic measurements, at various locations and tilts of the test sensor (Table 1 and Figure 2).

The data that most clearly reflected the geometry of the test object in the static and dynamic modes were extracted. The laboratory tests of the metrological characteristics of the low-cost handheld mobile scanner involved a juxtaposition of two sets of 3D data:

- MandEye static mode;
- MandEye dynamic mode;
- reference data from Leica ScanStation P40.

The compared datasets had the maximum object geometry scene from two sessions and diversified measurement modes.

### 2.2. MandEye Device—LIVOX: Technical Specifications and Optical Analysis

**Figure 3:**
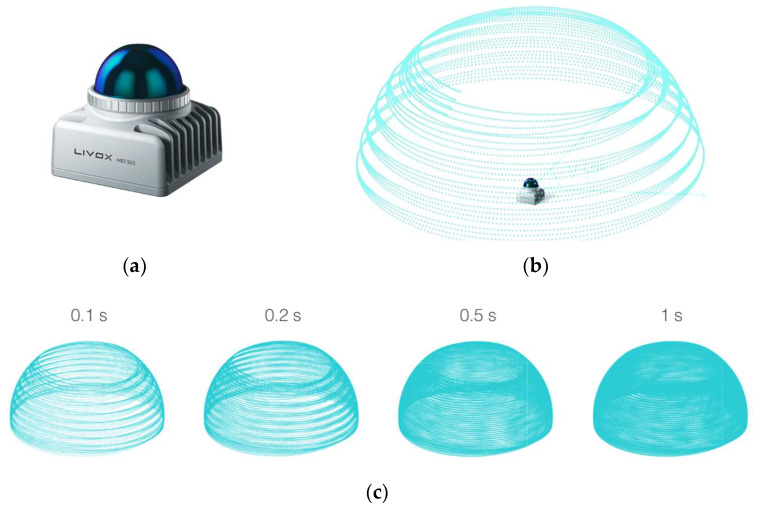
The Livox Mid-360 sensor: (**a**) the sensor; (**b**) scanning process; (**c**) point cloud patterns of the Livox Mid-360 accumulated over different integration times; source: Livox Mid-360 User Manual v1.2, 2024.

The test device is a mobile handheld laser scanner, MandEye, with open software and the Livox Mid-360 sensor (Livox, Livox Technology Company Limited Co., Hong Kong https://www.livoxtech.com) (Figure 3). Mid-360 is the latest generation of the Livox LiDAR for low-speed robotics. Unlike conventional mechanical LiDAR systems, it is powered by Livox’s rotating mirror hybrid-solid technology (Table 2) [https://www.livoxtech.com/mid-360 (accessed on 15 May 2024) [36]].

**Figure 4:**
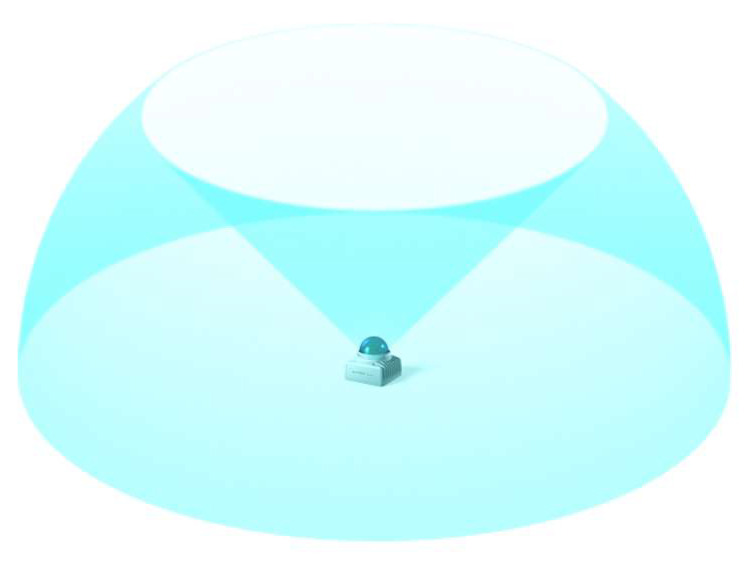
Measurement Range of the Scanner; source: https://www.livoxtech.com/mid-360 (accessed on 15 May 2024) [36].

The MandEye device, utilizing the Livox Mid-360 LiDAR scanner (Livox, Livox Technology Company Limited Co., Hong Kong https://www.livoxtech.com), operates at a laser wavelength of 905 nm, ensuring optimal parameters for a wide range of measurement applications while maintaining user safety (Class 1 according to IEC 60825-1:2014). The Livox Mid-360 employs advanced non-repetitive scanning technology, enabling high point cloud density and broader field-of-view (FOV) coverage, which is 360° horizontally and 59° vertically (Figure 4). The effective detection of objects depends on their position within the FOV; the detection range is shorter near the upper edge and longer near the lower edge. The measurement accuracy can reach up to 2 cm at a distance of 10 m, though this value may be influenced by environmental factors such as temperature and humidity, which can affect both accuracy and the device’s lifespan. Additionally, the Livox Mid-360 is equipped with an overheating protection mechanism and an automatic self-heating function for extremely low temperatures, allowing for operation across a wide temperature range from −20 °C to 55 °C, which is crucial for ensuring reliability in various environmental conditions [https://www.livoxtech.com/mid-360 (accessed on 15 May 2024) [36]].

### 2.3. Reference Data

**Figure 5:**
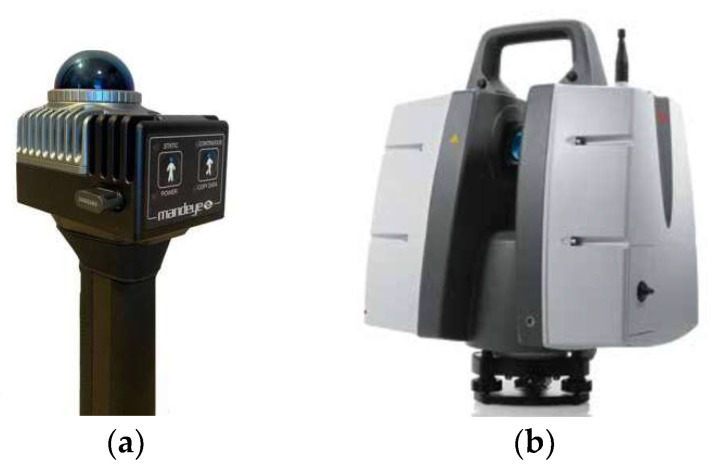
The measuring suite: (**a**) MandEye MLS, source: www.datcap.eu (accessed on 1 July 2024 ) [39]; (**b**) Leica P40 TLS, source: www.leica-geosystems.com (accessed on 5 July 2024) [38].

The 3D reference data for the tests (Figure 5a) were captured with a terrestrial laser scanner Leica ScanStation P40 (Figure 5b). Leica ScanStation P40 has a distance accuracy of 1.2 mm + 10 ppm, a point accuracy of 3 mm at 50 m horizontal and 6 mm at 50 m vertical, and high-quality noise removal at the stage of capturing TLS data, with a Root Mean Square (RMS) error of 0.4 mm at 10 m horizontal and of 0.5 mm RMS at 50 m vertical. This RMS value represents the standard deviation of the differences between the measured and true values, providing an indication of the accuracy of the noise reduction at the specified distances [38].

### 2.4. Scanning Outcomes

The scans yielded three cloud datasets that are different in terms of the number of points, capture time, and measurement mode. The consistencies among point clusters in the investigated data are compared below and divided into the top view (Table 3), side view (Table 4), isometric view, and cross-section view (detail) (Table 5). In a quantitative analysis, one should note both the degree of object geometry representation and the level of measurement noise, including room continuity artifacts (Table 4: Side view).

### 2.5. Evaluation of MLS Point Cloud Properties

#### 2.5.1. Sampling Resolution

**Figure 6:**
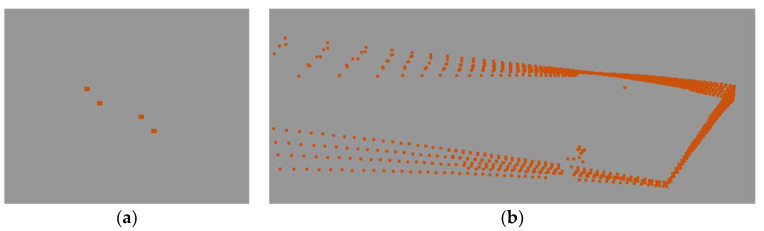
Basic measurement series: data sampling: (**a**) the smallest part of the measurement, four measurement lines, the first measured points; (**b**) a single measurement series.

Using timestamps, points from one scanning cycle were filtered out. In this way, it was able to determine horizontal and vertical sampling resolutions. As the scanner head employs non-repetitive scanning technology, consecutive scanning cycles improve the point cloud density over time (Figure 3c and Figure 6).

Angular sampling resolution: based on point coordinates (and station coordinates as X = 0.00, Y = 0.00):

- The vertical sampling resolution between consecutive lines is 3.0 degrees;
- The horizontal sampling resolution between consecutive points is 3.5 degrees.

#### 2.5.2. Observation Values for Points in the Cloud

**Figure 7:**
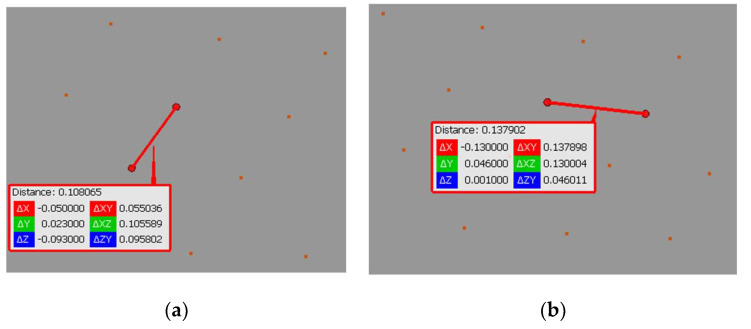
Sampling resolution: (**a**) vertical; (**b**) horizontal.

The tests were conducted on vertical planes (Figure 7) located at 6, 13, and 35 m from the scanner. A sample of points from a single scanning session was tested (basic sampling: Figure 6b). The mean values are shown in Table 6.

We then analyzed the cloud point density for the sampling resolution of a one-second measurement in the static mode (Table 7) (following the scanning process diagram in Figure 3c).

The sampling resolution and cloud density increase over the scanning duration and with a growing number of scanning sessions. The density and sampling resolution are unlimited for an object.

#### 2.5.3. Noise Analysis: Cloud Point ‘Width’

**Figure 8:**
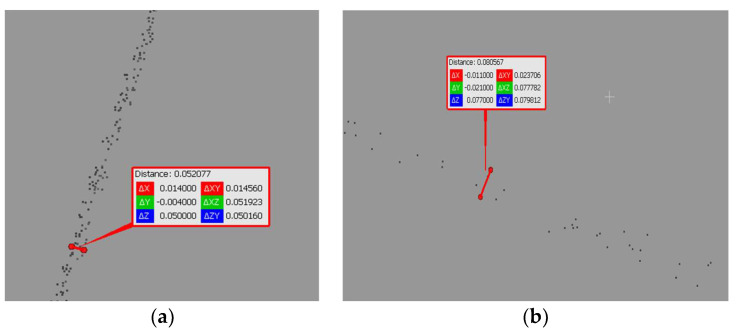
Noise analysis for an object at (**a**) 5 m; (**b**) 15 m.

We separated a 0.2 m slice from the point cloud to determine the measurement noise. Then, we measured the mean ‘width’ of the cloud point on smooth wall faces at 5 m and 15 m from the scanner. The results are 0.015 m and 0.024 m, respectively (Figure 8).

#### 2.5.4. Scan Noise—Invalid Points

**Figure 9:**
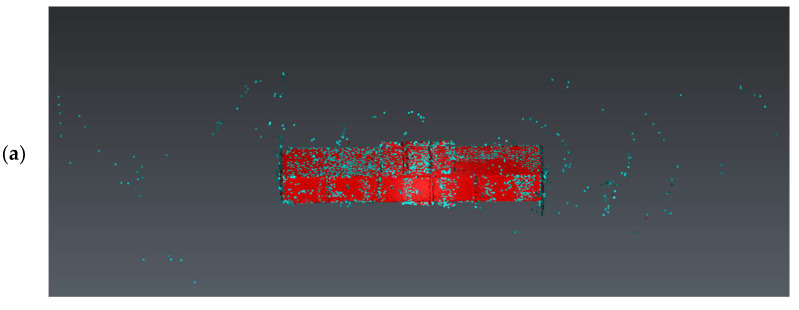

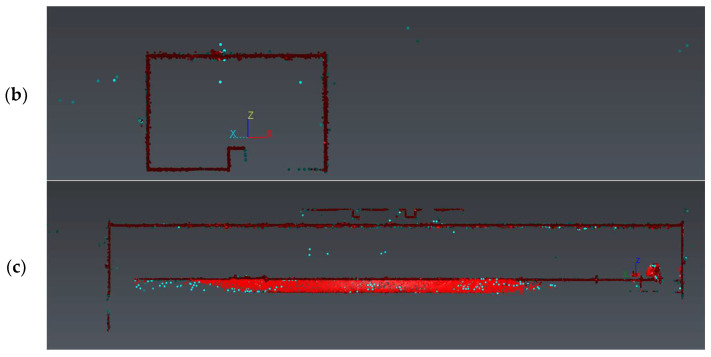
Noise distribution (blue) in relation to the point cloud (red) for the (**a**) isometric view of the object, (**b**) long-section of the object, and (**c**) cross-section of the object.

The analysis of noise and outlier distribution demonstrated that the share of points considered invalid amounted to 0.2% of the total number of cloud points (Figure 9). The identification of noise both inside and outside the room was conducted using an automatic filtering algorithm that took into account both the local density of points and their distances from neighboring points. This algorithm was based on distance-based filtering, which effectively detected and removed points that were significantly distant from others, suggesting they were erroneous measurements or noise. The process was fully automated, but users had the option to adjust key parameters, such as the maximum allowable distance between points, to precisely define the criteria for noise identification. As a result, the algorithm automatically removed the points considered to be noise, significantly improving the quality and reliability of the final point cloud.

#### 2.5.5. Identification of Target Points

**Figure 10:**
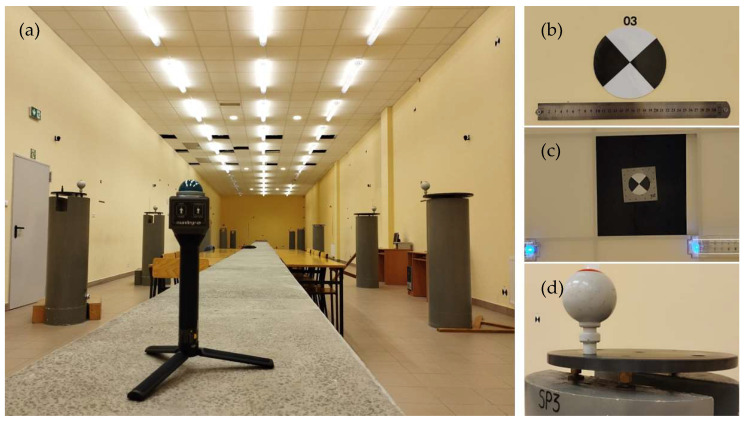
MandEye scans: (**a**) static mode; well-defined points in the space of the object (**b**) black and white targets on the walls; (**c**) black and white targets on the ceiling; (**d**) reference spheres.

The test of determination of well-defined points in the space of the scanned object (Figure 10a) involved black and white targets on the laboratory’s walls (Figure 10b) and ceiling (Figure 10c) and reference spheres (Figure 10d) positioned at regular intervals on single eccentric seats on the forced-centering pedestals. The point of the test was to verify whether it was possible to identify the centers of the black and white targets and white spheres, taking into account the geometry of the latter. The identification of targets points was accomplished by automatically detecting targets’ means in Leica Cyclone software version 9.1.

#### 2.5.6. Consistency of Geometrical Data and References

Assessing the geometrical consistency with the reference involved comparing spatial data captured with the low-cost scanner with a reference point cloud for geometric objects such as a cylinder, wall, or corners. The Authors analyzed the accuracy of oval, flat, and edge geometry for the test area by extracting a fragment of the point cloud from MandEye static mode and MandEye dynamic mode data and juxtaposing them with the reference data from Leica ScanStation P40. The linear deviations for each compared pair were detected and used as the metric of consistency of the geometric data with the reference.

## 3. Results

### 3.1. Identification of Target Points

**Figure 11:**
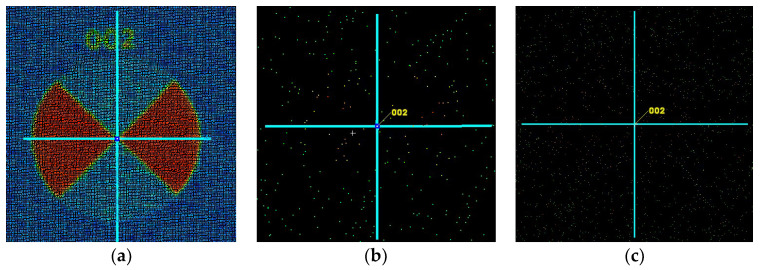
Identification of the geometric center of a black and white target T002: (**a**) Leica ScanStation P40, (**b**) MandEye, static mode, (**c**) MandEye, dynamic mode.

The identification of well-defined points, black-and-white targets, in the space of the scanned object demonstrated an 88% effectiveness for objects at 0–5 m from the MandEye scanner (static mode) (Table 8 and Figure 11). At more than 5 m, the spatial data were insufficient for estimating the center. The data from the dynamic mode were enough to determine the centers of 31% of the black-and-white targets (Table 8 and Figure 11c).

**Figure 12:**
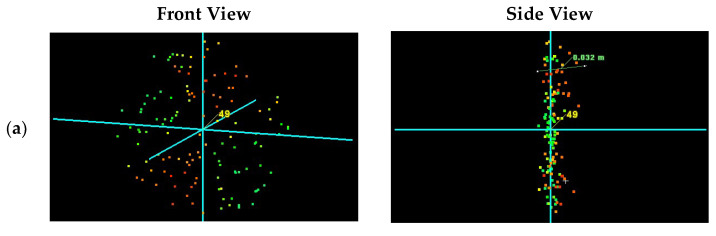

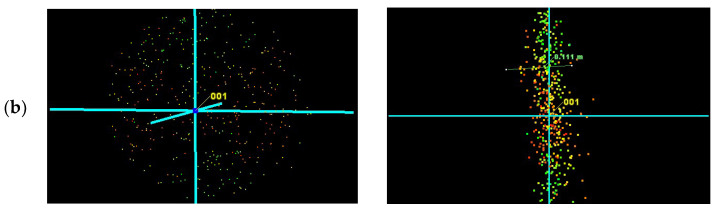
Identification of the geometric center of a black and white target: (**a**) MandEye, static mode, (**b**) MandEye, dynamic mode.

As shown above, well-defined points in the space of the scanned objects were identified nearly three times more often in the MandEye static scanning mode than in the dynamic mode. This is because, in this particular case, the data quality from the static mode exhibits less noise near the well-defined points (Figure 12).

**Figure 13:**
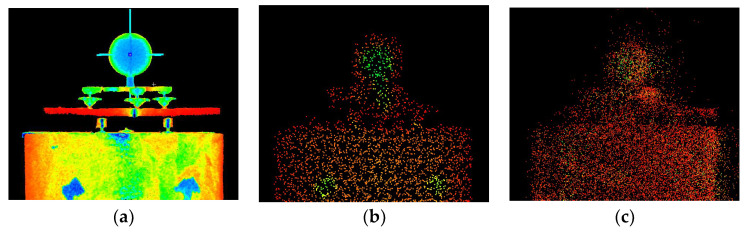
Identification of the geometric center of a reference sphere: (**a**) Leica ScanStation P40, (**b**) MandEye, static mode, (**c**) MandEye, dynamic mode.

The identification of well-defined points on the reference spheres in the space of the scanned objects was unsuccessful both in the static and dynamic modes (Figure 13). The noise level in the spatial data of the reference spheres from the low-cost laser scanner prevented estimating the center in clouds before and after filtering. The cloud representation of the reference sphere in the clouds from the manual scanner is an example of an artifact where the ghost effect affects the space outside and inside of the scanned object (Figure 13b,c).

The tie points identified in the Leica ScanStation P40 point clouds were the reference for registering the MandEye static mode and MandEye dynamic mode point clouds. Adjusting the point clouds yielded the mean absolute error for enabled constraints of ±0.013 m for the static dataset and ±0.017 m for the dynamic dataset. This level of geometrical consistency between the point clouds is a preliminary parameter for assessing the quality of the geometric accuracy of the low-cost handheld laser scanner.

### 3.2. Consistency of Geometrical Data and References

The assessment of the geometric consistency of scans from the low-cost MandEye laser scanner with the reference point cloud for cylindrical objects demonstrated a consistency of nearly 10 mm for the static mode and 15 mm for the dynamic mode (Table 9). Planar representation in both datasets indicated a consistency below 10 mm (Table 10). However, the MandEye static scan provided insufficient data for corners. In the mobile mode, the corner and planar consistency was 10 mm (Table 11).

## 4. Discussion

The affordability of sensors and the availability of open software for data capture and processing can be expected to drive the emergence of measuring solutions based on this technology [40,41]. Therefore, research on the metrological parameters of such solutions, both measuring instruments and mobile systems integrated with other sensors (IMU, GNNS, cameras), is highly relevant. The research aimed to complete laboratory measurements on a recently marketed low-cost mobile scanning system, the MandEye handheld MLS.

Our test results offer several conclusions and hypotheses regarding the potential applications of low-cost MLS with SLAM. Let us consider the completeness, quality, and accuracy of the data captured with the scanner. Regarding data completeness, the dynamic mode yielded negligible data voids compared to static solutions (Table 3, Table 4 and Table 5). This effect can also be expected in field conditions, such as during as-is surveys of structures or when constructing BIMs. The problem of data completeness and quality when using point clouds from various sources for structure surveys or BIM has been discussed by numerous authors [42,43,44,45].

Regarding data quality, although the nominal sampling resolution is relatively low (Table 6 and Table 7), the non-repetitive technology improves the point density rapidly so that such geometric features as surfaces and edges can be clearly identified. A more extended scan can densify the points to around a dozen times at a constant sampling rate, greater head repositioning, and using multiple scanning sessions. Combined with the noise of 0.015–0.024 m and flat and cylindrical surface representation at 0.010–0.015 m, it suggests that the device should be useful for modeling objects at lower levels of detail or for rapid surveys of architectural objects to calculate areas or volumes [46].

Data accuracy is yet another aspect to be considered. Although the mean absolute error of ±0.013 m for the static dataset adjusted to the reference point cloud confirms the accuracy parameters of the head declared by the manufacturer, the mean absolute error of ±0.017 m for the mobile datasets demonstrates that the trajectory adjustment algorithm is indeed effective. Obviously, this result is easy to achieve under laboratory conditions when the measurement path is short. Therefore, we intend to conduct further research in the field.

Considering the results, we propose a hypothesis that the tested device will be successfully used in areas where reduced data acquisition time compared to static scanning, a high level of geometrical data completeness to eliminate uncertainty for modeling and surveying construction and engineering structures, and centimeter-level geometric accuracy are essential. Such applications include the acquisition of data for various multivariate analyses [47], object modeling with LoD1, LoD2, and BIM accuracy [48], surveys of urban vegetation [49], and many others.

## 5. Conclusions

The laser scanner market is gaining momentum, offering new solutions at a rapid pace. Therefore, it is recommended that the methodology and algorithms for determining the measurement accuracy of such devices be investigated. Another argument for such research is that scanner manufacturers provide specifications for the accuracy of their devices in non-standardized ways or not at all. A methodology for testing accuracy parameters on a dedicated test field can be applied to various devices and facilitates the comparison of the results. There have been many scanners available commercially for several years. The test field can be used to investigate changes in the accuracy parameters of such devices between calibrations by the manufacturer.

The laboratory test filed at the Faculty of Environmental Engineering and Land Surveying of the University of Agriculture in Kraków for testing metrological characteristics of laser scanners is on par with the facilities reported in the literature and can be used for research identical to that described by the cited authors [1,3,6,7,8,9]. In addition, vast swaths of land at the University of Agriculture in Kraków (Poland) can accommodate a field test site for real-case field measurements, particularly considering the scanning range. Access to the university’s laboratory empowers our team to conduct research to devise original procedures and algorithms for testing the measurement accuracies of laser scanners. Furthermore, we have joined forces with other research centers and business stakeholders in Poland to investigate the accuracy of laser scanning measuring systems. We plan to conduct more extensive and exhaustive field tests of low-cost mobile laser scanners in the future.

## Figures and Tables

**Table 1:** Low-cost laser scanner test measurement variants.

Measurement Mode	Variants
MandEye, static mode	point cloud from a station on a concrete pedestal (close to SL1, SP1)
	point cloud from a station on a concrete pedestal (close to SL3, SP3, SL4, SP4); exposition close to the reference
	point cloud from a station on a concrete pedestal (close to SL7, SP7)
MandEye, dynamic mode	point cloud with a 0° tilt of the scanner, no thinning, and no manual adjustment
	point cloud with a 30° tilt of the scanner, no thinning, and no manual adjustment
Leica ScanStation P40	measurement with a resolution of 3 mm at 6 m on station SL3

**Table 2:** Livox Mid-360 specifications.

Laser wavelength	905 nm
Laser safety	Class 1 (IEC 60825-1:2014 [37]) (safe for eyes)
Detection range	40 m at 10% object reflectivity 70 m at 80% object reflectivity
Field of view	Horizontal: 360° vertical: −7~52°
Distance random error	≤2 cm at 10 m
Angular random error	≤0.15°
Point rate	200,000 points/s
Frame rate	10 Hz

Source: Livox Mid-360 User Manual v1.2, 2024, https://www.livoxtech.com/mid-360/downloads (accessed on 15 May 2024).

**Table 3:** Top view of the scan datasets.

(a) Leica ScanStation P40
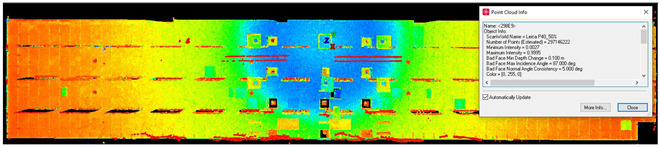
(b) MandEye, static mode
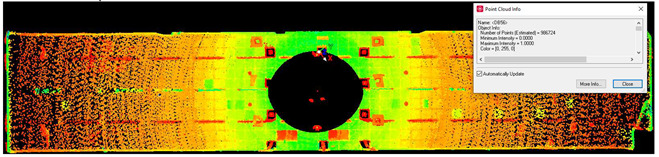
(c) MandEye, dynamic mode
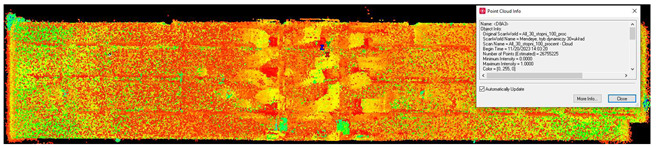

**Table 4:** Side view of the scan datasets.

(a) Leica ScanStation P40
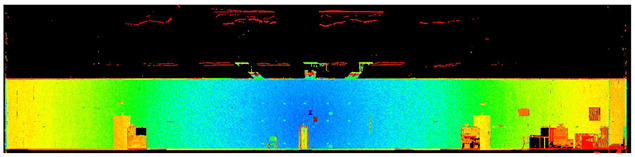
(b) MandEye, static mode
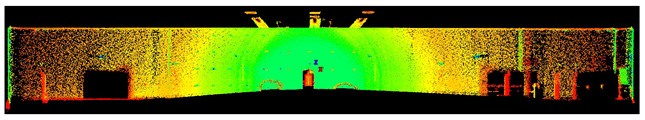
(c) MandEye, dynamic mode
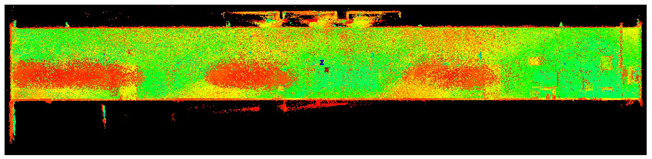

**Table 5:** Isometric view of the scan datasets and cross-section view (detail).

(a) Leica ScanStation P40	
** 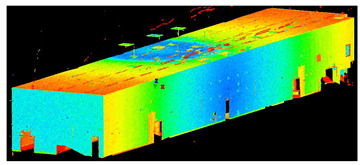 **	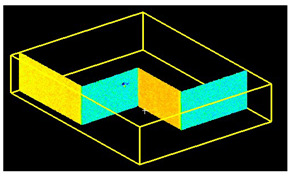
(b) MandEye, static mode	
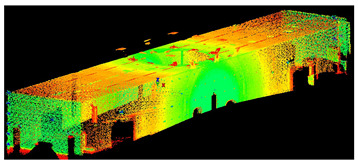	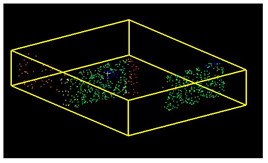
(c) MandEye, dynamic mode	
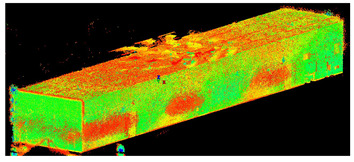	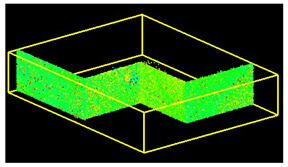

**Table 6:** Sampling resolution of a single measurement series (Figure 6b).

Distance [m]	6	13	35
Vertical resolution [m]	0.11	0.44	0.79
Horizontal resolution [m]	0.14	0.53	0.71

**Table 7:** Sampling resolution of the point cloud after one second of scanning in the static mode.

Distance [m]	6	13	35
Vertical resolution [m]	0.01–0.02	0.03–0.07	0.07–0.20
Horizontal resolution [m]	0.01–0.02	0.03–0.08	0.08–0.25

**Table 8:** Identification of reference targets with the scan data.

Target Distance [m]	Leica	MandEye	MandEye
	ScanStation P40	Static Mode	Dynamic Mode, 30°
0–5	100%	88%	31%
5–10	100%	-	
10–15	100%	-	
15–20	100%	-	
>20	100%	-	

**Table 9:** Assessment of dataset consistency with the reference for a cylindrical object.

Leica ScanStation P40 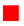 vs. MandEye, Static Mode 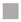	Leica ScanStation P40 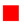 vs. MandEye, Dynamic Mode 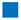
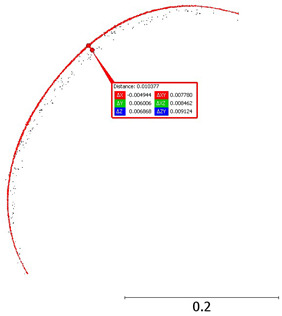	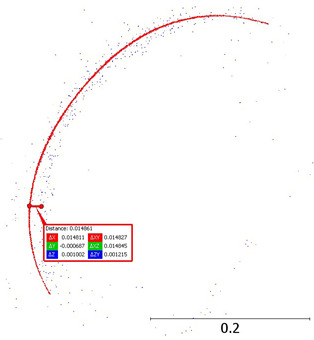

**Table 10:** Assessment of dataset consistency with the reference for a flat object.

Leica ScanStation P40 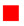 vs. MandEye, Static Mode 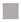	Leica ScanStation P40 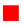 vs. MandEye, Dynamic Mode 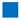
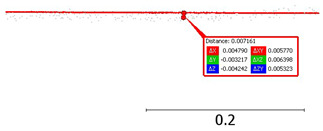	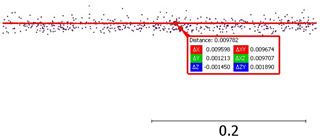

**Table 11:** Assessment of dataset consistency with the reference for an edge.

Leica ScanStation P40 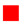 vs. MandEye, Static Mode 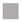	Leica ScanStation P40 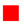 vs. MandEye, Dynamic Mode 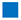
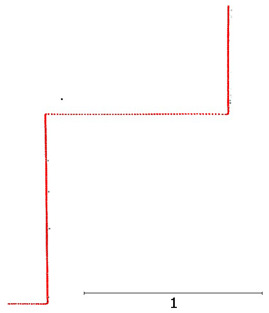	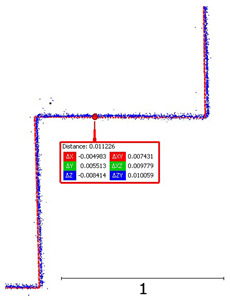

## Data Availability

The data used in the study are available from the corresponding author upon reasonable request.

## References

[1] Ortiz Arteaga A., Scott D., Boehm J. (2019). Initial Investigation of a Low-Cost Automotive Lidar System. ISPRS Int. Arch. Photogramm. Remote Sens. Spat. Inf. Sci..

[2] Glennie C.L., Kusari A., Facchin A. (2016). Calibration and Stability Analysis of the VLP-16 Laser Scanner. ISPRS Int. Arch. Photogramm. Remote Sens. Spat. Inf. Sci..

[3] Glennie C.L., Hartzell P.J. (2020). Accuracy Assessment and Calibration of Low-Cost Autonomous Lidar Sensors. ISPRS Int. Arch. Photogramm. Remote Sens. Spat. Inf. Sci..

[4] Glennie C. (2012). Calibration and Kinematic Analysis of the Velodyne HDL-64E S2 Lidar Sensor. Photogramm. Eng. Remote Sens..

[5] Wu W., Li J., Chen C., Yang B., Zou X., Yang Y., Xu Y., Zhong R., Chen R. (2023). AFLI-Calib: Robust LiDAR-IMU Extrinsic Self-Calibration Based on Adaptive Frame Length LiDAR Odometry. ISPRS J. Photogramm. Remote Sens..

[6] Hu T., Sun X., Su Y., Guan H., Sun Q., Kelly M., Guo Q. (2021). Development and Performance Evaluation of a Very Low-Cost UAV-Lidar System for Forestry Applications. Remote Sens..

[7] Hyyppä E., Kukko A., Kaijaluoto R., White J.C., Wulder M.A., Pyörälä J., Liang X., Yu X., Wang Y., Kaartinen H. (2020). Accurate Derivation of Stem Curve and Volume Using Backpack Mobile Laser Scanning. ISPRS J. Photogramm. Remote Sens..

[8] Lin J., Zhang F. Loam Livox: A Fast, Robust, High-Precision LiDAR Odometry and Mapping Package for LiDARs of Small FoV. Proceedings of the 2020 IEEE International Conference on Robotics and Automation (ICRA).

[9] Wang Y., Lou Y., Zhang Y., Song W., Huang F., Tu Z. (2021). A Robust Framework for Simultaneous Localization and Mapping with Multiple Non-Repetitive Scanning Lidars. Remote Sens..

[10] Cosarca C., Jocea A., Savu A. (2009). Analysis of Error Sources in Terrestrial Laser Scanning. RevCAD—J. Geod. Cadastre.

[11] Bosch T., Lescure M. (1995). Selected Papers on Laser Distance Measurement.

[12] Amann M.C., Bosch T., Lescure M., Myllyla R., Rioux M. (2001). Laser Ranging: A Critical Review of Usual Techniques for Distance Measurement. Opt. Eng..

[13] Seitz P. (2007). Photon-Noise Limited Distance Resolution of Optical Metrology Methods. Opt. Meas. Syst. Ind. Insp. V.

[14] Płatek A. (1995). Elektroniczna Technika Pomiarowa w Geodezji.

[15] Wanic A. (2007). Instrumentoznawstwo Geodezyjne i Elementy Technik Pomiarowych.

[16] Blais F. (2004). Review of 20 Years of Range Sensor Development. J. Electron. Imaging.

[17] Schulz T. (2007). Calibration of a Terrestrial Laser Scanner for Engineering Geodesy.

[18] Beraldin J.A., Blais F., Lohr U. (2010). Laser Scanning Technology. Airborne and Terrestrial Laser Scanning.

[19] Stilla U., Yao W., Jutzi B. (2007). Detection of Weak Laser Pulses by Full Waveform Stacking. Int. Arch. Photogramm. Remote Sens. Spat. Inf. Sci..

[20] Quintero M.S., Garcia J.L.L., Genechten B.V. (2008). 3D Risk Mapping Theory and Practice on Terrestrial Laser Scanning. Training Material Based on Practical Applications.

[21] Klapa P., Mitka B. (2017). Edge Effect and its Impact upon the Accuracy of 2D and 3D Modelling Using Laser Scanning. Geomat. Landmanag. Landsc..

[22] Pawleta M., Igielska A. (2009). Analiza Dokładności Wybranych Modeli Naziemnych Skanerów Laserowych Firmy Zoller + Frohlich GmbH.

[23] Lichti D.D., Stewart M.P., Taskiri M., Snow A.J. (2002). Benchmark Tests on a Three Dimensional Laser Scanning System. Geomat. Res. Australas..

[24] Boehler W., Bordas M., Marbs A. Investigating Laser Scanner Accuracy. Proceedings of the XIX CIPA Symposium.

[25] Pesci A., Teza G. (2008). Effects of Surface Irregularities on Intensity Data from Laser Scanning: An Experimental Approach. Ann. Geophys..

[26] Kersten T.P., Sternberg H., Mechelke K., Gruen A., Kahmen H. (2005). Investigations into the Accuracy Behavior of the Terrestrial Laser Scanning System Mensi GS100. Optical 3-D Measurement Techniques VII.

[27] Clark J., Robson S. (2004). Accuracy of Measurements Made with a CYRAX 2500 Laser Scanner against Surface of Known Colour. Int. Arch. Photogramm. Remote Sens. Spat. Inf. Sci..

[28] Mitka B. (2007). Możliwości Zastosowania Naziemnych Skanerów Laserowych w Procesie Dokumentacji i Modelowania Obiektów Zabytkowych. Arch. Fotogram. Teledetekcji.

[29] Pepe M., Alfio V.S., Costantino D., Herban S. (2022). Rapid and Accurate Production of 3D Point Cloud via Latest-Generation Sensors in the Field of Cultural Heritage: A Comparison between SLAM and Spherical Videogrammetry. Heritage.

[30] Thomson C., Apostolopoulos G., Backes D., Boehm J. (2013). Mobile Laser Scanning for Indoor Modelling. ISPRS Ann. Photogramm. Remote Sens. Spat. Inf. Sci..

[31] Stefano F., Chiappini S., Gorreja A., Balestra M., Pierdicca R. (2021). Mobile 3D Scan LiDAR: A Literature Review. Geomat. Nat. Hazards Risk.

[32] Ryding J., Williams E., Smith M.J., Eichhorn M.P. (2015). Assessing Handheld Mobile Laser Scanners for Forest Surveys. Remote Sens..

[33] Będkowski J. (2024). Open Source, Open Hardware Hand-Held Mobile Mapping System for Large Scale Surveys. SoftwareX.

[34] Software for Operating the Manndeye Scanner. https://github.com/JanuszBedkowski/mandeye_controller.

[35] Software for Generating Point Clouds from MLS. https://github.com/MapsHD/HDMapping.

[36] Mid-360 Scanner. https://www.livoxtech.com/mid-360.

[37] (2014). TC 76 Optical Radiation Safety and Laser Equipment. Safety of Laser Products—Part 1: Equipment Classification and Requirements.

[38] Leica Geosystems A.G. (2017). Leica Scanstation P30/P40 Product Specifications.

[39] DatCap Company, Sales Offer: MLS. www.datcap.eu.

[40] Pyae A., Lin N., Zaw Z. (2022). Open Source and Affordable Terrestrial Laser Scanner. Int. Arch. Photogramm. Remote Sens. Spat. Inf. Sci..

[41] Karam S., Vosselman G., Peter M., Hosseinyalamdary S., Lehtola V. (2019). Design, Calibration, and Evaluation of a Backpack Indoor Mobile Mapping System. Remote Sens..

[42] Skrzypczak I., Oleniacz G., Leśniak A., Zima K., Mrówczyńska M., Kazak J.K. (2022). Scan-to-BIM Method in Construction: Assessment of the 3D Buildings Model Accuracy in Terms of Inventory Measurements. Build. Res. Inf..

[43] Gawronek P., Makuch M., Mitka B., Bożek P., Klapa P. 3D Scanning of the Historical Underground of Benedictine Abbey in Tyniec (Poland). Proceedings of the International Multidisciplinary Scientific GeoConference Surveying Geology and Mining Ecology Management.

[44] Chen S., Fan G., Li J. (2023). Improving Completeness and Accuracy of 3D Point Clouds by Using Deep Learning for Applications of Digital Twins to Civil Structures. Adv. Eng. Inform..

[45] De Geyter S., Vermandere J., De Winter H., Bassier M., Vergauwen M. (2022). Point Cloud Validation: On the Impact of Laser Scanning Technologies on the Semantic Segmentation for BIM Modeling and Evaluation. Remote Sens..

[46] Kofman J., Borribanbunpotkat K. (2014). Hand-Held 3D Scanner for Surface-Shape Measurement Without Sensor Pose Tracking or Surface Markers: A Compact Hand-Held 3D Scanner Simultaneously Projecting Multiple Light Lines Is Presented, Enabling 3D Surface-Shape Measurement Without Requiring Sensor Tracking or Surface Markers. Virtual Phys. Prototyp..

[47] Mrówczyńska M., Skiba M., Sztubecka M., Bazan-Krzywoszańska A., Kazak J.K., Gajownik P. (2021). Scenarios as a Tool Supporting Decisions in Urban Energy Policy: The Analysis Using Fuzzy Logic, Multi-Criteria Analysis and GIS Tools. Renew. Sustain. Energy Rev..

[48] Liu J., Xu D., Hyyppä J., Liang Y. (2021). A Survey of Applications with Combined BIM and 3D Laser Scanning in the Life Cycle of Buildings. IEEE J. Sel. Top. Appl. Earth Obs. Remote Sens..

[49] Bauwens S., Bartholomeus H., Calders K., Lejeune P. (2016). Forest Inventory with Terrestrial LiDAR: A Comparison of Static and Hand-Held Mobile Laser Scanning. Forests.

